# Effect of Reminiscence on Cognitive Status and Memory of the Elderly People

**Published:** 2014

**Authors:** Golbahar Akhoondzadeh, Shamsolmamalek Jalalmanesh, Hamid Hojjati

**Affiliations:** 1 PhD Student, Department of Nursing, International Campus, Kerman University of Medical Sciences, Kerman, Iran.; 2 Department of Nursing, School of Nursing and Midwifery, Islamic Azad University, Tehran Branch, Tehran, Iran.; 3 Young Researchers Club, Islamic Azad University, Aliabad Katoul Branch, Aliabad Katoul, Iran

**Keywords:** Cognition, Elderly, Memory, Reminiscence

## Abstract

**Objective:** The present study aimed to determine the effect of reminiscence on cognitive status and memory of a sample of elderly people who lived in aged day centers in Golestan province, Iran.

**Methods:** This study was a semi-experimental research. The subjects consisted of 45 elderly people who referred to the aged day centers of Golestan province. Each four groups of 45 aged subjects (4 groups of 10-12 subjects) took part in 8 reminiscence sessions which lasted one to one and half hour. Cognitive status and of the aged, before and after taking part, was assessed by Mini Mental State Examination and Wechsler Memory Scale.

**Results: **Mean (±SD) cognitive status score at pre-test stage was 24 (±2) which increased to 25 (±2) at post-test stage (p < 0.01). Mean (±SD) intelligence quotient was 87 (±7) at pre-test stage which increased to 92 (±10) at post-test stage (p < 0.01).

**Conclusion:** Reminiscence sessions held for the aged studied here had beneficial effects on cognition and memory of the subjects.

## Introduction

Aging leads to physiological, social and mental changes (1). According to the World Health Organization (WHO), aged is an individual who is older than 60 years (2). As the sanitation has improved in recent decades, the number of the aged increases in the world (3); and in near future the number of the 65 years old people and more will increase. The number of the elderly in the world is estimated to be 82 million by the year 2050 and by that time the estimated rate of elderly will be 37.5%. This figure was 17% in 2005 (1). Statistical indicators show that the process of becoming old in Iran has started as well and it is predicted that a ten year age will be added to average age of Iran in a 20-year time from 1996 to 2017 (4). According to the central information of the United Nations in Tehran, the number of elderly in Iran will reach to 26 million and 393 thousand people, who are 26% of the whole population of the nation. According to the mentioned statistics, it can be stated that under the present circumstances Iran is passing the young population to elderly and will soon join the countries with elderly population (3, 4). 

People gradually lose some of their mental states, social states and physiological actions while this loss may not cause them to be dependent, but it will have some enormous effect on their vulnerability (5). Cognitive status disturbance is a prevalent problem of the elderly that has devoted a vast field of aged disturbance to itself; so that about 35% of the aged show different aspect of cognitive disturbances among which Alzheimer’s disease is the most typical example (6). When people get about 70, some unstable aspect of amnesia is observed in their intelligence quotient. Though the aged preserve their abilities to understand the subjects and their position; but they may have difficulties with giving rapid and precise responses (7). 

In 2012, Simon demonstrated that cognitive treatments cause enhancement of cognitive status and memory in affected people (8). Group reminiscence is a type of cognitive treatment. This is an opportunity for the elderly to balance their social and occupational position by expressing memories (9). Recalling the issues and events accompanied by practice and experience can reinforce the memory and preserve cognitive status of elderly people (6, 10). This method is an effective treatment for cognitive functions and reinforces the effect of the other therapeutic methods. It particularly reinforces the effect of anti-dementia medicines in the aged who are at the risk of amnesia (11). Despite the worldwide spread use, inexpensiveness and easiness, the use of reminiscence in our country -where the number of elderly is continuously increasing- is not common and not a lot effort has been put on this method in Iranian culture (4). 

This study was done due to limited reports on this topic in the literature and almost no report from Iran. We intended to assess the effect of group reminiscence on the cognitive status of the elderly people.

## Materials and Methods

In this semi-experimental study including pretest and posttest, the subjects who aged more than 60 years and referred to two aged day centers (Mirdamad in Gorgan and Farzanegan in Ghonbad Kavoos) in Golestan province in north-east of Iran were recruited through convenience sampling method. The studied aged people included elderly ones with at least the ability of reading and writing and making verbal and eye contact and had no previous record of chronic psychiatric disorders and also had no experience of “death of a loved one” in hospitals, no critical emotional trauma such as the death of a close relative during the past three months and also were not addicted to any type of substance or alcohol. 

First, the cognitive status of the aged was studied using the Mini-Mental State Examination (MMSE) and the ones whose scores were less than 20 were excluded. This questionnaire is one of the most common measurement instruments which tests cognitive status in the fields of navigation, recording, attention, calculating, reminding and designing and finally yields a total score (12). If the test shows no problem in any of the above fields, the score will be 30, which is the maximum possible score, but the scores less than 20 indicate that the cognitive disabilities are deeper. The scores between 20 and 25 indicate that the existence of cognitive trauma is trivial (13). Frooghian et al. reported Cronbach’s alpha of this scale as 0.78 and using a cutoff point of 21, reported its sensitivity as 90% and its specify as 84% (14).

The Standard Wechsler Memory Scale was used to assess the memory quotient. Wechsler Memory Scale in form “A” consists of personal awareness about routine issues, recognition, awareness of the time and location, mental control, logical memory, repeating words forward and backward, expressing memory and learning associations. The intelligence quotient (IQ) score was obtained using obtained score from this test and adding the corrected score of the age group. The validity with the Cronbach’s alpha coefficient for the subscales is as the following: general information 0.9, logical memory 0.89, digit span 0.77, sight rebuilding 0.83, and verbal association 0.81 (15). In Moradi study, Cronbach’s alpha figures for the subscales of general information, navigation, mental control, logical memory, digit span, sight rebuilding and verbal association were 0.75, 0.70, 0.65, 0.63, 0.65, 0.69, and 0.70, respectively (16). Validity of the questionnaire was calculated by means of content validity and structure validity (15).The validity of these two questionnaires (Wechsler memory and Mini Mental Status Examination) has been confirmed by expert psychiatrists and neurologists and has frequently been used, both in national and foreign studies to measure the cognitive status and memory. 

Four groups of 48 aged subjects (10-12 subjects in each group) took part in the present study. Averagely, the time length of the research was 2 (±0.7) years. Any subject who did not attend more than one session was excluded. Three subjects were excluded for this reason. The required permissions were received from the authorities at the most beginning and all the involved aged voluntarily took part in this study and they were all asked.

After filling in the cognitive status questioners before the intervention, its score was considered as a pre-test score and after intervention the score was considered as the posttest score. Then eight reminiscence sessions were held, each session lasted 45 to 60 minutes twice a week and it was held for 4 weeks. The researcher conducted all the sessions of this study. All the reminisce sessions based on telling the childhood, youth time, education, marriage and forming family, children birth, occupational experiences, successes, travels, celebrations, particular and important past memories of the aged were the main subjects they were talking about. After finishing the reminiscence sessions, the questionnaires were completed once again by the help of the therapists. The scores obtained here were considered as the post-test scores (Table 1).

The data were analyzed using the SPSS for Windows 16.0 (SPSS Inc., Chicago, IL, USA). After testing the distribution of the data by the Kolmogorov-Smirnov test, for comparing the averages before and after the intervention, the paired t-test with the confidence interval 95% was used.

## Results

Mean (±SD) age of the subjects was 70 (±6) years old, 62% of them were males (n = 28) and 38% were females (n = 17). 

Sixty-two percent (n = 28) were married and 38% (n = 17) were widows or widowers. Mean (±SD) number of children was 5 (±2). Regarding the job, 29% (13 people) were homemakers and 28% (12 people) were farmers and laborers. Seventy-one percent (32 people) were illiterate.

Mean (±SD) cognitive status score, based on MMSE, at pre-test stage was 24 (±2) which increased to 25 (±2) at post-test stage (p < 0.01) 

Mean (±SD) intelligence quotient, according to the Wechsler Memory scale, was 87 (±7) at pre-test stage which increased to 92 (±10) at post-test stage (p < 0.01).

## Discussion

The results showed that reviewing life memories causes improvement and enhancement of cognitive status of the aged. Akauma stated that group reminiscence is a conceptional method for investigating and reviewing the life incidents and believes that this method which stimulates the memory and reinforces the excitement effects can cause improvement of mental status (17). Namati Dehkordy et al. believed that group reminiscence through the reduction of agitation and the promotion of cognitive status causes the stimulation and reinforcement of elderly people memory (18). Amini et al. believed that the rehabilitation methods in the elderly cause memory improvement and the functional status of the old people (19). The results of Aquar study showed that this method is an effective treatment for cognitive improvement and reinforces the effects of other treatment methods in elderly people (11). Woods et al. showed that reminiscence along with memory stimulation prevents cognitive functional destruction. He believed that this method is an effective treatment in improving patients suffering from dementia (20).

**Table 1:** Content of the group reminiscence sessions

**First session**	Introducing the process and group purpose and getting familiar with other members of the group
**Second session**	Telling the memory of the childhood by the aged
**Third session**	Talking about the youth time and studying
**Fourth session**	Giving some information about marriage and birth of their children
**Fifth session**	Stating the memories and occupational experiences and their success in that period of time
**Sixth session**	Telling the memories of the celebrations and travels of their past
**Seventh session**	Presenting the memories related to particular and important events in their lives
**Eighth session**	Adding up and finishing the sessions

Joosten-Weyn Banningh et al. believe that cognitive treatment causes enhancement of awareness and memory. It also results in increased motivation in receiving care on behalf of patients suffering from cognitive recognition (21). Lin et al. propounded the effect of this method on cognitive status in his study (22). Chang et al. stated that group reminiscence can improve cognitive status in addition to functional reinforcement and daily activities of patient (23). 

Recalling the memories is usually a conceptional method for investigating and reviewing life incidents and is known as a mental process in which memory stimulation and cognitive status are the aim of discussion (11). In England, group reminiscence has been considered as a treatment for patients suffering from dementia and is carefully applied on elderly people living in elderly adult day centers (21). Peng et al. stated that behavior and cognitive treatments such as reminiscence is an inexpensive, useful and effective method to improve depression and cognitive disorders such as dementia in elderly people and reinforces medical therapeutic effects. In addition to mental status improvement, this method has some positive effects on physical status of the elderly (24). Mackinlay and Trevitt believed that memory recalling and reviewing causes the person to reach his/her personal identity, feels joyful, and abating sadness, anxiety and feeling of sin (25). Westerhof et al. mentioned that group reminiscence causes recalling reinforcement and improves mental status (26). Thorgrimsen et al. reported that reminiscence along with memory stimulation prevents destruction of functional cognitive. They also believe that this method is an effective treatment to manage patients suffering from dementia (27). Fujiwara et al. noted that group reminiscence along with compatibility reinforcement and individual skills cause awareness and increases social functions, life satisfaction, self-respect and reduces solitude feeling in the aged. This finally leads to reinforcement and promotion of elderly routine life (28). Therefore, this method of treatment which is simple, inexpensive and applicable can be used in all health care centers for the elderly. It is also suggested that health care centers can take an effective step towards maintenance and promotion of mental status of the elderly by introducing this method to families (4). One of the limitations of this study was the lack of a control group, so it is suggested that other researchers do the experimental study along with two control groups. 

## Conclusion

Regarding the growth of the aged population in Iran and beneficial effects of group reminiscence on improving cognitive status in the elderly, it is suggested that authorities and those who take care of elderly and families endeavor to employ this easy, cheap and applicable method.

## Notes

**Declaration of interest:** None.
